# The association between human endogenous retroviruses and multiple sclerosis: A systematic review and meta-analysis

**DOI:** 10.1371/journal.pone.0172415

**Published:** 2017-02-16

**Authors:** Elena Morandi, Radu Tanasescu, Rachael E. Tarlinton, Cris S. Constantinescu, Weiya Zhang, Christopher Tench, Bruno Gran

**Affiliations:** 1 Division of Clinical Neuroscience, University of Nottingham School of Medicine, Nottingham, Nottingham, United Kingdom; 2 Division of Clinical Neurosciences, University of Medicine and Pharmacy Carol Davila, Department of Neurology, Colentina Hospital, Bucharest, Romania; 3 University of Nottingham School of Veterinary Medicine and Science, Nottingham, United Kingdom; 4 Department of Neurology, Nottingham University Hospitals NHS Trust, Nottingham, United Kingdom; 5 Division of Rheumatology, Orthopaedics and Dermatology, University of Nottingham School of Medicine, Nottingham, United Kingdom; Charite Universitatsmedizin Berlin, GERMANY

## Abstract

**Background:**

The interaction between genetic and environmental factors is crucial to multiple sclerosis (MS) pathogenesis. Human Endogenous Retroviruses (HERVs) are endogenous viral elements of the human genome whose expression is associated with MS.

**Objective:**

To perform a systematic review and meta-analysis and to assess qualitative and quantitative evidence on the expression of HERV families in MS patients.

**Methods:**

Medline, Embase and the Cochrane Library were searched for published studies on the association of HERVs and MS. Meta-analysis was performed on the HERV-W family. Odds Ratio (OR) and 95% confidence interval (CI) were calculated for association.

**Results:**

43 reports were extracted (25 related to HERV-W, 13 to HERV-H, 9 to HERV-K, 5 to HRES-1 and 1 to HER-15 family). The analysis showed an association between expression of all HERV families and MS. For HERV-W, adequate data was available for meta-analysis. Results from meta-analyses of HERV-W were OR = 22.66 (95%CI 6.32 to 81.20) from 4 studies investigating MSRV/HERV-W (MS-associated retrovirus) envelope mRNA in peripheral blood mononuclear cells, OR = 44.11 (95%CI 12.95 to 150.30) from 6 studies of MSRV/HERV-W polymerase mRNA in serum/plasma and OR = 6.00 (95%CI 3.35 to 10.74) from 4 studies of MSRV/HERV-W polymerase mRNA in CSF.

**Conclusions:**

This systematic review and meta-analysis shows an association between expression of HERVs, and in particular the HERV-W family, and MS.

## Introduction

Multiple Sclerosis (MS) is a chronic demyelinating disease of the central nervous system (CNS) and one of the most common causes of neurological disability in young adults, with a higher incidence in women than men [1]. Among environmental factors able to trigger MS pathogenesis on a background of genetic susceptibility, viral infections are of particular relevance. In addition to herpesviruses, such as HHV-6, VZV, and especially EBV [2], the expression of Human Endogenous Retrovirus (HERVs) has been considered as a risk factor for developing MS and for disease progression [3].

**Fig 1:**
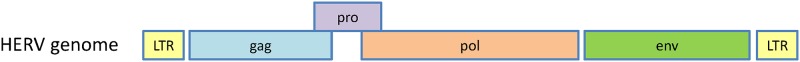
Genetic structure of HERVs. LTRs (Long Terminal Repeat) regions bound the genome with four major viral genes: *gag* (encoding matrix and retroviral core), *pol* (reverse transcriptase and integrase), *pro* (protease), and *env* (envelope).

HERVs originate from exogenous infectious retroviruses that integrated into cells of the germ line 70 to 30 million years ago and came to represent almost 8% of the human genome. Over time, HERVs have generally lost their original capacity to retro-transpose or reinfect, having accumulated a series of mutations and recombination events. HERVs are multicopy families with each family consisting of many different loci in the human genome. They are classified into 31 families ranging in copy number from one to many thousands. These families are classified by a naming system on the basis of the tRNA specificity of the primer binding site, corresponding to the amino acid that would be added to the HERV were it translated into viral proteins (HERV-W,-K,-H etc.) [4]. HERVs have generally maintained the same genetic structure as exogenous retroviruses. Two LTRs (Long Terminal Repeat) regions bound the genome with four major viral genes: *gag* (encoding matrix and retroviral core), *pol* (reverse transcriptase and integrase), *pro* (protease), and *env* (envelope) (Fig 1).

The first HERV reported to be associated with MS in the late 1980s was the Multiple Sclerosis-associated retrovirus (MSRV), a member of the HERV-W family [5].

In addition to HERV-W, an increased expression of HERV-K and HERV-H families in the blood, brain or cerebrospinal fluid (CSF) from people with MS has also been reported by some groups [6], but not others [7].

The literature on this topic has been confused by a number of issues. The original studies on MSRV/HERV-W [5, 8] assumed that functional viral particles were involved and focussed on detection of cell-free (presumably virion associated) RNA. The later realisation that none of the 213 HERV-W loci in the human genome are fully replication competent cooled enthusiasm for the hypothesis of retroviral involvement in MS [9]. Reports of an association between MSRV/HERV-W sequences and MS however continued, some affirming the association, some refuting it. Further confusion arose from these reports due to the plethora of detection methods (PCR and protein based), patient cohorts and sample types (blood, central nervous system, cell free and cell based) analysed (presented in this systematic review) and the variety of names given to the sequences detected. Recent detailed analysis of reported HERV-W/MSRV/Syncytin-1 sequences has demonstrated that they originate from a mosaic of loci and it is unlikely that the methods used to date are able to distinguish those from a single locus [9].

Subsequent to the initial reports of HERV association with MS, it has become clear that HERVs perform, at least in some cases, physiological roles in their hosts, with some HERV-W loci able to produce envelope proteins, in particular the syncytin-1 protein involved in human placental fusion [10]. These proteins have also been demonstrated to have immunomodulatory effects in experimental models [11]. It is also now clear that the promoter and enhancer elements in the proviral LTRs of endogenous retroviruses can and do act as transcriptional regulatory networks and affect the transcription levels of genes they are inserted in or near. In the case of HERV-W this is at least 55 genes, some previously associated with neurological disease [9].

The role of HERVs in MS both from an epidemiological association and a pathogenic mechanism point of view is therefore still a debateable topic. This systematic review therefore reviews the available data on the expression of different HERV families in MS patients and the methods used. In addition, we perform a meta-analysis of the expression of HERV-W *env* and *pol* RNA in MS patients and controls to resolve the epidemiological association of HERVs with MS

## Methods

In performing this study, we followed the PRISMA (Preferred Reporting Items for Systematic reviews and Meta-Analyses) protocol [12]. The PRISMA checklistis given in Supporting Information files (S1 Table). Details of the protocol for this systematic review were registered on PROSPERO (registration number CRD42016047290) and can be accessed at http://www.crd.york.ac.uk/PROSPERO/#index.php

### Data source and search

Medline, Embase and the Cochrane Library up to 9th September 2016 were searched using the keywords “MULTIPLE SCLEROSIS” AND (“HERV” OR “HERVS” OR “HERV-W” OR “HERV-H” OR “HERV-K” OR “MSRV”) with no time restriction. Only papers published in English were included. Two independent investigators (EM and RTan) extracted the data from the literature databases and the references cited in the identified papers.

### Inclusion criteria

Full text articles were included in the systematic review if they were case-control studies containing data on the expression of viral proteins or RNA or DNA of any HERV family, in any type of tissue, from patients diagnosed with MS and from control groups (either healthy or pathological). Studies were further sub-grouped based on the retroviral family, the techniques employed, the protein/nucleic acid identified, and the type of tissue.

A subset of these articles was used for a quantitative meta-analysis if they studied the same protein or nucleic acid in the same type of tissue, using the same techniques. An additional inclusion criterion for the meta-analysis was the use of the same type of control group (healthy subjects or other controls). We performed a meta-analysis only if a minimum of 4 comparable studies met these criteria. Reviews and conference abstracts were excluded.

### Data extraction

EM and RTan independently screened the papers yielded by the search to eliminate duplicates and to verify that inclusion criteria were met. Any disagreement was resolved through discussion with a third investigator (BG). The articles extracted were first organised in five different groups on the basis of the HERV families studied. From each eligible study the first author, the year of publication, the protein/nucleic acid investigated, the type of tissue analysed, the techniques used, the populations studied (number of cases and controls, number of women, mean age, source and type of disease), the country of origin, and a summary of the results were extracted independently by EM and RTan. Such results included the percentage or number of cases and controls considered positive for HERV expression, the presence or absence of an association and its statistical significance, as expressed by the authors, and any correlations with disease course, if available. The data extracted focused on the number of cases with HERV-W expression rather than the level of expression as too few studies reported quantitative expression levels for analysis.

### Meta-analysis

The association of different viral proteins/nucleic acids with MS was analysed separately.

The odds ratio (OR) were estimated by the Mantel-Haenszel test and the standard error (SE) and the 95% confidence intervals (CI) were calculated using SPSS (IBM, version 22). We calculated the total OR, the p-value of fixed effects (inverse of variance) and of random effects (DerSimonian-Laird test) using StatsDirect (Version 2.8.0). We evaluated the heterogeneity calculating the Inconsistency (I^2^) and the Cochrane Q p-value. Egger’s regression test was used to examine publication bias.

### Quality assessment

The quality assessment of included studies was based on the Newcastle-Ottawa assessment scale (NOS) [13].

## Results

### Study selection

**Fig 2:**
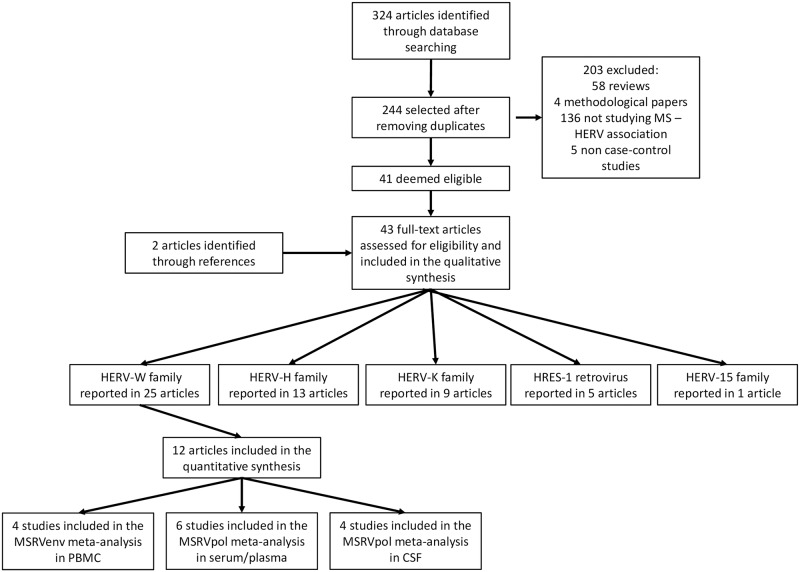
Flow chart of the study selection and procedure. Articles identified and selected for the qualitative and quantitative synthesis.

The online search identified 324 articles (Fig 2). After removing the duplicates, 244 articles were retained. A further 203 articles (58 reviews, 4 methodological papers, 136 articles that did not study the association between HERVs and MS, and 5 studies that were not case-control studies) were excluded. Forty-one papers were deemed eligible and 2 additional articles were identified from the references of the selected articles.

The 43 papers included were categorised into 5 groups based on the HERV family studied (S2, S3, S4 and S5 Tables) and are presented in the Table 1. Six papers studied more than one HERV family and were included in more than one table. Twenty-five articles focused on HERV-W; 13 on HERV-H; 9 on HERV-K, whilst HRES-1 was studied in 5 and HERV-15 in 1 paper.

**Table 1:** Summary of the countries in which different research groups investigated specific HERV families referenced in our study.

COUNTRY	HERV-W	HERV-H	HERV-K	HRES/ HERV-15
*CANADA*	Antony 2007 [7]	Antony 2006 [28]	Antony 2006 [28]	
	Antony 2006 [28]	Antony 2004 [33]	Antony 2004 [33]	
	Antony 2004 [33]	Johnston 2001 [34]	Johnston 2001 [34]	
	Johnston 2001 [34]			
*BRAZIL*	do Olival 2013 [16]			
*DENMARK*	Brudek 2009 [26]	Nexo 2016 [39]	Nexo 2016 [39]	Rasmussen 2000 [50]
		Nissen 2012 [42]		Rasmussen 1999 [51]
		Laska 2012 [41]		Rasmussen 1998 [53]
		Hansen 2011 [40]		Rasmussen 1997 [45]
		Nexo 2011 [38]		Rasmussen 1996 [52]
		Brudek 2009 [26]		
		Christensen 2003 [37]		
		Christensen 2000 [36]		
*FRANCE*	Van Horssen 2016 [32]		Rasmussen 1997 [45]	
	Perron 2012 [19]			
	Perron 2005 [31]			
	Menard 1997 [22]			
	Garson 1998 [8]			
	Perron 1997 [5]			
*GERMANY*	Schmitt 2013 [35]			
	Laufer 2009 [29]			
*POLAND*	Zawada 2003 [24]			
	Nowak 2003 [25]			
*ITALY*	Mameli 2009 [10]			Martinelli-Boneschi 2012 [54]
	Arru 2007 [17]			
	Mameli 2007 [18]			
	Dolei 2002 [20]			
	Serra 2001 [21]			
*SPAIN*	Garcia-Montojo, 2014 [14]	de la Hera 2014 [43]	De la Hera 2013 [48]	
	Garcia-Montojo, 2013 [15]	Alvarez-Lafuente 2008 [30]		
	Alvarez-Lafuente 2008 [30]			
*SOUTH AFRICA*	de Villiers 2006 [23]			
*UK*			Moyes 2008 [47]	
			Moyes 2005 [46]	
*USA*			Tai 2008 [49]	

Examining all the publications found, we could perform three meta-analyses, in which at least 4 studies of association between HERV and MS reported the same viral targets, tissue samples and techniques compared to the same type of control group (either healthy or neurological controls). Four studies investigated RNA expression of the HERV-W sequence “MSRV/HERV-W*env*” by RT-PCR (Reverse transcriptase—polymerase chain reaction) in peripheral blood mononuclear cells (PBMC) from patients with MS and HC, 6 studies investigated RNA expression of the HERV-W sequence “MSRV/HERV-W*pol*” detected by RT-PCR in the serum or plasma from MS patients and HC, and 4 studies investigated RNA expression of the HERV-W sequence “MSRV/HERV-W*pol*” detected by RT-PCR in the CSF from MS patients and OND (Other Neurological Disease) controls. The remaining 25 studies were not included in the meta-analysis because they did not meet the inclusion criteria.

### HERV-W

The literature search identified 25 articles reporting on an association between HERV-W and MS by 9 different research groups (Table 1, S2 Table).

#### HERV-W in peripheral blood

Expression of the MSRV/HERV-W viral proteins or RNA *env* and p*ol* in the blood (PBMC or serum/plasma) was reported in 20 publications by Sardinian, French, Polish, Spanish, Brazilian, South African, Danish, German, and Canadian groups. In 15 studies MSRV/HERV-W*env* [10, 14–19] or MSRV/HERV-W*pol* [5, 8, 17, 20–25] RNA or protein were found to be increased in serum/plasma or PBMC of MS patients compared to control groups (HC or OND) by different techniques [RT-PCR, Flow cytometry (FC) and ELISA].

Two studies demonstrated different levels of expression of the MSRV/HERV-W*env* in different types of blood cells [26, 27]. In these studies MSRV/HERV-W*env* RNA and protein expression detected by RT-PCR and FC were increased in monocytes, NK and B cells, but not in CD4^+^ and CD8^+^ T cells of MS patients compared to controls [26, 27]. Three studies (from Canada and Germany) did not detect increased levels of HERV-W*env* RNA (either MSRV/HERV-W or syncytin-1 that share 94% sequence identity at RNA level) in plasma, total PBMC and cell subtypes from MS patients compared to control groups [7, 28, 29].

#### HERV-W in the CSF

Six papers studied the expression of HERV-W in the CSF from MS patients and controls. MSRV/HERV-W*pol* and *env* RNA detected by RT-PCR were found to be over-expressed in people with MS compared with HC and OND by the French [5] and Italian groups [17, 20]. The Spanish [30] and Canadian groups [7, 28] did not find an increased expression of HERV-W*env* RNA by RT-PCR in MS patients.

#### HERV-W in the brain

Nine publications studied the expression of MSRV/HERV-W in the brain tissue. A French and an Italian group showed the presence of MSRV/HERV-W Pol, Gag and Env proteins and RNA in infiltrating macrophages clustered around endothelial cells in MS lesions [18, 19, 31, 32], but not in the brain of HC or OND using immunohistochemistry and RT-PCR. By contrast, using the same techniques, a Canadian group found an increased expression of HERVWE1*env* RNA (syncytin-1) but not MSRV/HERV-W*env*, in the brains of MS patients compared to OND [7, 28, 33, 34]. A German group used Next Generation Sequencing (NGS) to detect the expression of HERV-W loci in the brain, without significant differences between HC and MS [35].

#### HERV-W in different types of MS

Three studies in MS patients and HC looked at MSRV/HERV-W expression in different forms of MS. The French group found differences between Relapsing-remitting (RR-), primary-progressive (PP-) and secondary-progressive (SP-) MS with an increase of MSRV/HERV-W*env* DNA copy number, but not of RNA and protein expression, in the progressive forms of MS [19]. The Spanish group detected a higher expression of MSRV/HERV-W*env* RNA in SPMS than RRMS [14]. Patients with an elevated MSRV/HERV-W*env* DNA copy number had a higher degree of disability, according to their Expanded Disability Status Scale (EDSS) score [15].

### MSRV/HERV-W meta-analysis

For our meta-analysis of the association of MSRV/HERV-W with MS, 12 articles were suitable for inclusion according to the criteria described in the Methods. Together, these studies included 478 MS patients, 330 HC and 145 OND. The characteristics of participants in the included studies are summarised in Table 2. A separate meta-analysis was performed for each viral protein (*env* and *pol*) and for each different tissue. Four studies investigated MSRV/HERV-W*env* RNA in PBMC (111 MS patient and 58 HC), 6 studies investigated MSRV/HERV-W*pol* RNA in serum/plasma (309 MS patients and 272 HC), and 4 studies investigated MSRV/HERV-W*pol* RNA in CSF (187 MS patients and 145 HC).

**Table 2:** Characteristics of participants in the included studies.

Study ID	HERV	SAMPLE	METHOD	MS CASES							CONTROLS				COUNTRY
				TOT	SEX	MEANAGE	SOURCE	RRMS	SPMS	PPMS	TOT	SEX	MEAN AGE	SOURCE	
***Env expression in PBMC***															
S. do Olival 2013 [16]	*MSRV env*	PBMC	RT-PCR	10			Clinically definite MS				10			HC with no familiar history of MS	Brazil
Perron 2012[19]	*MSRV env*	PBMC	RT-PCR	58			Hospital Neurologic. Depart.				26	Sex-matched	Age-matched	Healthy blood donors transfusion centre	European
Mameli 2007[18]	*MSRV env*	PBMC	RT-PCR	35		37.1	Active MS				14		32.3	Blood donors transfusion centre	Sardinia
Mameli 2009[10]	*MSRV env*	PBMC	RT-PCR	8	F 4	43	Depart. of Neurosc. University Sassari	5	1	2	8			6 Blood donors and 2 Health Care Operators	Sardinia
***Pol expression in serum/plasma***															
Arru 2007[17]	*MSRV pol*	PLASMA	nested PCR	147	F 102	33.8	Sardinia, Ferrara Pamplona Stockholm				98	Sex-matched	Age-matched	HC from Sardinia and Spain	European (Sweden, Spain, Italy, Sardinia)
Dolei 2002[20]	*MSRV pol*	PLASMA	RT-PCR	39	F 25	36.9	Sardinian origin, free of IT for at least 3 months	24	4		39		37	Healthy blood donors from transfusion centre of Sassari	Sardinia
Serra 2001[21]	*MSRV pol*	PLASMA	RT-PCR	25		36.6	Sardinian origin Neurology Clinic, free of IT for at least 3 months	15	2		25		37.6	Healthy blood donors without known MS risk	Sardinia
Garson 1998[8]	*MSRV pol*	SERUM	RT-PCR	17			French clinically active MS				36			Healthy UK adults	France
de Villiers 2006[23]	*MSRV pol*	SERUM	RT-PCR	49			South African MS patients of European descent	37	7	5	39		Age-matched	Healthy blood donors and laboratory personnel from same ethnic group	South Africa (European descendent)
Nowak 2003[25]	*MSRV pol*	SERUM	RT-PCR	32		Median 37.7	Clinically definite untreated				27		Median 34.5	Healthy adults	Poland
***Pol expression in CSF***															
Alvarez-Lafuente 2008[30]	*HERV-W*	CSF	RT-PCR	48	F 34	32.6	First clinically evident demyelinating event	48			44	F 29	OIND 43.7 ONIND 39.4	23 OIND 21 ONIND	Spain
Arru 2007[17]	*MSRV pol*	CSF	nested PCR	98			Sardinia, Ferrara Pamplona Stockholm				81	Sex-matched	Age-matched	OND from Sardinia, Ferrara Stockholm	European (Sweden, Spain, Italy, Sardinia)
Dolei 2002[20]	*MSRV pol*	CSF	RT-PCR	31		36.7	Sardinian origin, free of IT for at least 3 month				10		31.9	4 CNS OIND, 6 CSF ONIND	Sardinia
Perron 1997[5]	*MSRV pol*	CSF	RT-PCR	10	F 6	34.7	Grenoble Paris Milan	2	3	5	10	F 7	36.7	Grenoble Paris Milan	France

Abbreviations: MSRV, MSRV/HER-W; pol, Polymerase; env, Envelope; PBMC, Peripheral Blood Mononuclear Cells; CSF, Cerebrospinal Fluid; RT-PCR, Reverse Transcription Polymerase Chain Reaction; MS, Multiple Sclerosis; HC, Healthy Control; OND, Other Neurological Disease; RRMS, Relapsing-Remitting MS; SP, Secondary-Progressive MS; PP, Primary-Progressive MS; F, Female; OIND, Other Inflammatory Neurological Disease; ONIND, Other Non-Inflammatory Neurological Disease; IT, Immunomodulatory Treatments

**Fig 3:**
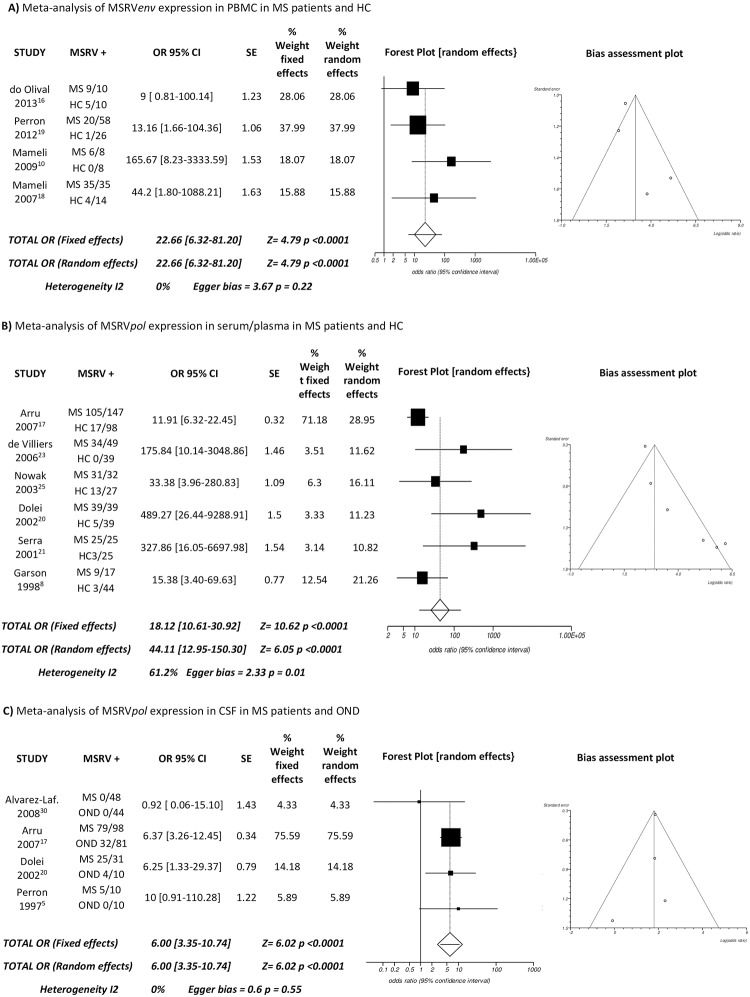
Summary meta-analysis. Forest plot and bias assessment funnel plot of comparison of the expression of (A) MSRV/HERV-W*env* in PBMC, (B) MSRV/HERV-W*pol* in serum/plasma, and (C) MSRV/HERV-W*pol* in CSF in MS patients vs controls.

Seventy of 111 MS patients (63%) and 10 of 58 HC (17.2%) expressed MSRV/HERV-W*env* in PBMC with an OR of 22.66 (95%CI 6.32–81.20; p<0.0001 for fixed and random effects, 0% inconsistency and Egger test for publication bias p = 0.22). Two-hundred forty-three of 309 MS patients (78.6%) and 41 of 272 HC (15%) expressed MSRV/HERV-W*pol* in plasma/serum with an OR of 18.12 (95%CI 10.61–30.92; p<0.0001 for fixed effects) and OR of 44.11 (95%CI 12.95–150.30; p<0.0001 for random effect, inconsistency 61.2% and Egger test for publication bias p = 0.0088). One-hundred nine of 187 MS patients (58%) and 37 of 145 OND (25%) expressed MSRV/HERV-W*pol* in CSF with an OR of 6.00 (95%CI 3.35–10.74; p<0.0001 for fixed and random effects, 0% inconsistency and Egger test for publication bias p = 0.55) (Fig 3).

#### Quality assessment

In the NOS scale of the quality assessment of the studies included in the meta-analysis the maximum score that could be achieved by a study was 10 stars. The majority of the studies scored less than half of the maximum score, with a mean of 4.5 (Table 3). The highest scoring study was Perron et al. 2012 with score of 8 stars [19]. With regards to selection criteria, most studies had adequate definitions of cases and controls. The comparability of cases and controls was good for 6 studies that matched at least the age (Table 3). For the exposure criteria, all the studies used appropriate molecular techniques; 3 studies reported blinding sample analysts (Table 3). No studies reported a non-response rate (Table 3).

**Table 3:** Quality assessment of included studies.

	*Selection*				*Comparability*		*Exposure*			TOT
STUDY ID	S1	S2	S3	S4	C1	C2	E1	E2	E3	
S. do Olival 2013 [16]	x			x			x			3
Perron 2012 [19]	x	x	x		x	x	xx	x		8
Mameli 2009 [10]	x	x	x				xx	x		6
Mameli 2007 [18]	x		x		x		x			4
Arru 2007 [17]	x	x			x	x	xx	x		7
Dolei 2002 [20]	x		x		x		x			4
Serra 2001 [21]	x	x	x	x	x		x			6
Garson 1998 [8]			x				x			2
de Villiers 2006 [23]			x		x	x	x			4
Nowak 2003 [25]			x				x			2
Alvarez-Lafunte 2008 [30]		x	x				x			3
Perron 1997 [5]		x	x		x	x	x			5

Abbreviations: S1 case definition; S2 representativeness cases: defined hospital, over period of time, defined area; S3 selection controls: community controls; S4 definition controls: no history of MS; C1 matched for age; C2 matched for other factor; E1 ascertainment of exposure (x, appropriate detection technique; xx, appropriate detection technique and blinding); E2 same method for case and controls; E3 non-response rate

### HERV-H

Thirteen articles from 3 different research groups from Spain, Denmark and Canada focused on the association between HERV-H and MS (Table 1, S3 Table).

The presence of HERV-H in MS samples was detected for the first time by Christensen in 1998 [36]. The expression of HERV-H*env* and *gag* RNA by PCR was increased in the serum and PBMC of Danish MS [36, 37]. B cells and monocytes, but not T cells, from patients with active MS expressed higher levels of HERV-H Env protein detected by FC when compared to patients with stable MS and to controls [26].

In contrast, no difference in RNA expression was detected by CSF PCR between Spanish MS patients and neurological controls [30]. Three studies did not find differences in the expression of HERV-H*env* RNA in the brain [33] and PBMC [28] and HERV-H*gag* RNA in the brain [34] between Canadian MS and OND patients.

The Danish group [38, 39] identified a SNP (single nucleotide polymorphisms) (rs391745) on HERV-Fc1 (a HERV-H retrovirus on chromosome X) that was associated with RRMS and SPMS in the Danish and Norwegian populations [40]. They showed an increased expression of HERV-Fc1*gag* RNA in plasma and of HERV-Fc1Gag protein in T cells and monocytes in active MS patients compared to non-active MS and HC [41]. The same group did not find differences in the HERV-Fc1*gag* DNA copy number in PBMC between the MS and HC groups [42].

A systematic review summarized the presence of HERV-Fc1 in the Spanish, Danish and Norwegian MS cohorts [43]. The authors found an initial OR of 1.17 (p = 0.004; 95%CI: 1.05–1.30) in favour of the association which increased to 1.27 (95%CI: 1.11–1.45) after the Spanish cohort was excluded, bringing the heterogeneity from 82% to 0% [40].

### HERV-K

Nine studies published by 6 different research groups in Denmark, France, Canada, UK, USA and Spain describe an association between MS and the HERV-K family (Table 1, S4 Table). More specifically, the studies focussed on the HERV-K10,-K115,-K113 and–K18 loci of this family. HERV K alleles (in particular the HERVK-113 and the HERV-K 115 loci) are known to be polymorphic in the human population (e.g., they are absent in some individuals) [44] which is an additional factor that may affect expression of these sequences in MS.

The first report in 1997 by a French group found no difference in the expression of HERV-K10*env* RNA in the PBMC and brain samples in MS patients compared to HC [45]. The Canadian group reported an increased expression of HERV-K*pol* [34], but not of HERV-K*env* [28, 33] in the brain of MS patients compared to control groups, using RT-qPCR. A British group used DNA PCR and showed an increased frequency of HERV-K113, but not HERV-K115, in MS and Sjogren`s syndrome compared to HC in a British population [46]. The same authors could not reproduce the result in a larger study using unaffected parents of MS patients as control group [47], but a Danish group found an association between the SNP rs2435031 near HERV-K113 and MS [39].

A meta-analysis studying the link between HERV-K18 polymorphisms on chromosome 1 and autoimmune diseases found an association between the haplotype HERV-K18.3 (97Y-154W) and the American and Spanish MS population with an OR of 1.22 (95%CI:1.09–1.38) [48, 49]. A stronger association was detected in the subgroup of MS patients carrying the HLADRB1* 15:01 risk allele, known as a genetic risk factor for MS [48].

### HRES and HERV-15

Rasmussen et al. in Denmark produced the 5 publications dealing with the relationship of the human T cell leukemia virus-related endogenous sequence (HRES) with MS (Table 1, S5 Table). These authors reported an association between MS and different haplotypes of HRES-1 defined on the basis of a SNP. They detected an increased frequency of haplotype 1 in British [50] and haplotypes 2 and 3 in Danish MS patients [51, 52], but no haplotype association in Chinese MS patients [53] compared to HC. There was no difference in the level of RNA expression of HRES-1 between Danish MS patients and HCs [45].

A genome-wide association study (GWAS) in progressive MS identified a locus on chromosome 7q35 (rs996343(G)) that resides in a retroviral element of the HERV-15 family [54] (S5 table).

## Discussion

In this study we demonstrate an association between MS and HERVs.

The HERV-W family has been studied in most detail since the discovery of its member MSRV/HERV-W in biological samples obtained from MS patients [5]. This is a multi-copy gene family, of which most loci are truncated or lack open reading frames. In the human genome there are at least 213 copies [9]. We performed a qualitative and quantitative analysis of the available data on the association between HERV-W expression and MS. Overall, 20 of 25 articles that studied MSRV/HERV-W in MS report an association between MS and increased expression of MSRV/HERV-W in blood, CSF, and brain tissue. For the meta-analysis we had aimed at identifying retroviral family-specific articles that studied the presence or absence of the same protein or nucleic acid in the same type of tissue, using the same techniques. An additional inclusion criterion for the meta-analysis was the presence of the same control group. We decided to perform a meta-analysis only if a minimum of 4 studies met these criteria. On this basis, a quantitative meta-analysis could only be performed for a) HERV-W family MSRV/HERV-W*env* detected in PBMC of MS and HC by RT-PCR b) MSRV/HERV-W*pol* detected in serum or plasma of MS and HC by RT-PCR and c) MSRV/HERV-W*pol* detected in CSF of MS and OND by RT-PCR.

All the meta-analyses that we performed showed a strong association between MSRV/HERV-W*pol* and MSRV/HERV-W*env* and MS.

Using healthy blood donors as control group, the results of the *env* meta-analysis showed a high OR (22.66) with no inconsistency or publication bias. High OR were obtained from the *pol* meta-analysis in serum/plasma as well, but a high heterogeneity between studies (61.2%) and evidence of publication bias were noted. Genetic differences between the studied populations could underlie such heterogeneity, at least in part. The study with the largest population in the *pol* meta-analysis was a multicentre study including patients from Sweden (32%), Spain (41%) and Sardinia (27%) [17], while the studies with very high OR, but a relatively small population, were only from Sardinia [20, 21], an Italian island with peculiar genetics and a high incidence of MS [55]. Other important confounders could be sex, age and source of case and controls. Unfortunately due to limitations in the information provided by these articles (age, sex, type of MS, and source), it was not possible to stratify the population based on these parameters.

In line with the results found in the blood comparing MS and HC, the *pol* meta-analysis in CSF of MS and OND showed strong OR (6.00) as well, with no inconsistency or publication bias. This suggests that MSRV/HERV-W*pol* expression is specifically associated with MS rather than general neurological diseases.

The major limitations of these meta-analyses are the relatively small population samples included in the analysis, which might not be representative of the whole population of patients with MS or controls, and the small number of studies included. The preponderance of papers from one Sardinian research group is a potential confounding factor in this meta-analysis, however the Sardinian cohort does not represent the majority of cases in each meta-analysis and all meta-analyses included studies from multiple groups and geographical locations with consistent results. There are regional genetic differences in MS prevalence with Sardinia having a very high incidence of the disease [56] and this may be reflected in the association of MS with HERV-W. However, it is clear that the association is found in more than just this one ethnic group. What is not clear despite the inclusion of one Brazilian cohort in the meta- analysis is whether this association holds true for all non-European populations.

Of all the articles studying HERV-W, only 4 were included in the meta-analysis for MSRV/HERV-W*env* in PBMC, 6 for MSRV/HERV-W*pol* in serum/plasma and 4 for MSRV/HERV-W*pol* in CSF. Only one of the publications with negative results was eligible for the quantitative analysis [30], while all the others did not meet the inclusion criteria, creating a potential bias. Specifically, Antony et al 2006 [28] and 2007 [7], Schmitt et al 2013 [35] and Laufer et al 2009 [29] did not report data that could be interpreted as number of MSRV/HERV-W-positive and–negative MS and control subjects, but presented them as relative expression and often reporting only the mean or the p value for the differences. We decided not to establish arbitrary cut-off values to identify “positive” and “negative” samples because we cannot assume that every author would be able and willing to provide us with original data. On the other hand, positive/negative cut-off values are not explicitly presented in the publications included in the meta-analysis. For these reasons, we only used the data as they are actually presented in the peer-reviewed articles we have selected. Such papers were included in the qualitative, rather than quantitative analysis.

Among negative studies, Antony et al reported an association between MS and syncytin-1 rather than MSRV/HERV-W*env*. Syncytin-1 only describes the Env protein encoded by ERVWE-1, a replication-incompetent sequence on chromosome 7q21-22. At the RNA level the reported MSRV/HERV-W*env* sequence and syncytin-1 share 94% sequence identity, only differing for a 12-nucleotide insertion, making their discrimination difficult.

What is now clear is that the PCR-based methods used for detecting MSRV/HERV-W or Syncytin loci detect a mosaic of sequences rather than specific loci making such analysis difficult to interpret [9]. Differences in real-time PCR methods [57] may also contribute to such complexity, which will require further clarification. PCR is the most commonly used method for HERV detection, but it does have limitations, including the inclusion or exclusion of different loci from the same group of viruses depending on the design of the primers used, which vary between studies (S6 Table). For the serum and PBMC studies included in the meta-analysis, we did not find a correlation between the primers used and the OR of the study. In the CSF, however, the only study that used a different set of primers reported the lowest OR (Alvarez Lafuente 2008 [30], S6 Table). This underlies the difficulty in establishing which HERV loci are responsible for differences observed between studies. Moreover, creation of recombinants between different sequences in vitro may also lead to detection of RNAs that do not originate from genomic DNA. It cannot be excluded that future improvements in detection techniques will lead to the discovery of additional HERVs associated with MS, but potentially also to disproving previously reported significant associations [35].

The issue of HERV-W complexity is also extended to studies of HERV protein expression. It often remains unclear as to which genomic locus the observed HERV proteins are derived from and therefore, whether the precise identity of the protein recognized by the MSRV/HERV-W Env antibodies can be established. A further set of potentially confounding factors are the reports of variation of DNA proviral copy number in the CNS tissue of MS and control patients [15, 19]. Given that HERV-W cannot function as a retrovirus capable of increasing its own copy number by de novo insertions there may be other factors involved, such as genetic variation in the presence or absence of certain HERV-W loci. This has not been previously reported for HERV-W, but does occur for other retroviral families such as HERV-K [44]. It is also possible that other mechanisms of duplication of transposons (such as LINE retrotransposition) are at play.

The majority of the studies scored poorly (<5) in the NOS scale for quality assessment. This could be related to poor reporting rather than poor conduct (for example only 3 of 12 studies specified to use the same technique for cases and controls). Difficulties in the enrolment of community controls matched with at least two factors (age and sex) with MS patients could be a real bias to take in consideration in the analysis.

Overall, the association between MSRV/HERV-W and MS is strong. In addition to an association with a diagnosis of MS, the expression of MSRV/HERV-W has also been associated with the occurrence of disease progression.

From a mechanistic point of view, MSRV/HERV-W Env can activate Toll like receptor-4 (TLR4) and induce the production of iNOS and proinflammatory cytokines such as TNFα, IL-1β and IL-6, with associated reduction in oligodendrocyte differentiation capacity and myelin protein production [11, 58–60]. Furthermore, MSRV/HERV-W Env has immunostimulatory properties, as demonstrated by its capacity to substitute for mycobacterial lysate as a component of complete Freund’s adjuvant (CFA) in MOG_35-55_-induced EAE in C57-BL/6 mice, leading to full-blown disease [11]. Such evidence suggests a possible role of MSRV/HERV-W in the pathogenesis of MS. It is possible, however, that the expression of MSRV/HERV-W in MS patients could be a consequence rather than a cause of inflammation, as suggested by increased expression of MSRV/HERV-W by monocytes stimulated by TNF-α [34].

The fact that herpesviruses can activate MSRV/HERV-W [27] suggests that EBV, an infectious environmental factor strongly associated with MS, could induce higher MSRV/HERV-W expression and underlie at least in part the association of HERVs with MS. Epigenetic regulation, escape from viral restriction factors, and other as yet unknown mechanisms could also explain this increased transcriptional activity in MS.

In addition to the HERV-W family, studies on HERV-H, HERV-K, HRES and HERV-15 also showed an association with MS in the qualitative analysis.

For these HERV families, 17 of 28 studies reported a significant association between HERV expression and MS, whereas the other 11 studies did not. More limited agreement between these studies may be explained by genetic differences among populations [52, 53], differences in the patterns of expression of different HERVs in CNS or peripheral tissues at different stages of MS [28], and by methodological issues. Technical issues were raised with regards to the adequate storage conditions of the samples [30] or the RT-PCR methodology used [57].

Unfortunately, for HERV families different from HERV-W, there are fewer than 4 studies meeting the mentioned inclusion criteria, therefore a quantitative analysis was not possible. With regards to HERV-Fc1 (H family, included in our qualitative analysis), a meta-analysis was already published by De la Hera et al 2014 [43], only focussing on the strength of association between the presence of the rs391745 SNP and MS.

In conclusion, our findings strongly support the evidence for an association between HERVs, and in particular MSRV/HERV-W, and MS. Further studies are required to better understand the nature and pathophysiology of this association.

## Supporting information

S1 Table PRISMA checklist. (DOC)

S2 Table Studies included in the HERV-W qualitative analysis: 25 articles studying the association between HERV-W and MS by 9 different research groups. (DOCX)

S3 Table Studies included in the HERV-H qualitative analysis: 13 articles studying the association between HERV-H and MS by 3 different research groups. (DOCX)

S4 Table Studies included in the HERV-K qualitative analysis: 9 articles studying the association between HERV-K and MS by 6 different research groups. (DOCX)

S5 Table Studies included in the HRES and HERV-15 qualitative analysis: 5 articles studying the association between HRES and MS by 1 research group. 1 article studying the association between HERV-15 and MS by 1 research group.
(DOCX)

S6 Table Primers used in the studies included in the HERV-W meta-analysis. (DOCX)
