# Supplementary material for: The association between human endogenous retroviruses and multiple sclerosis: A systematic review and meta-analysis
Source: PLoS One. 2017 Feb 16;12(2):e0172415. doi: 10.1371/journal.pone.0172415 (PMC5313176; doi:10.1371/journal.pone.0172415)
Supplement: S2 Table — (DOCX) [file pone.0172415.s002.docx]

S2 Table

| **HERV** | **SAMPLE** | **TECHNIQUE** | **POPULATION IN THE STUDY** | **COUNTRY** | **RESULT** | **STUDY** |
| --- | --- | --- | --- | --- | --- | --- |
| MSRVenv RNA | PBMC | RT-PCR | MS (112) HC (68) | SPAIN | Increased expression of MSRVenv  in MS (p=0.004) | ***Garcia-Montojo, 2014^14^*** |
| MSRVenv DNA copy number | PBMC | RT-qPCR | MS (178) HC (124) | SPAIN | Increased copy number of MSRVenv DNA in MS (p=4.17e^-7^) | ***Garcia-Montojo, 2013^15^*** |
| HERV-W DNA/RNA | CSF | RT-PCR | MS (48) inflammatory OND (23) no inflammatory OND (21) | SPAIN | Expression of HERV-W RNA and DNA detection MS 0% inflammatory OND 0% no inflammatory OND 0% | ***Alvarez-Lafuente 2008^30^*** |
| HERV-W LOCI | BRAIN | RT-PCR NGS | MS (6) HC (7) | GERMANY | Overall very similar relative transcript levels between MS and control brain tissue samples | ***Schmitt 2013^35^*** |
| HERV-Wenv RNA | PBMC | RT-PCR | MS (4) HC (4) | GERMANY | No significant difference in the relative cloning frequencies of the different HERV-W env loci | ***Laufer 2009^29^*** |
| HERV-Wenv RNA | PBMC | RT-qPCR | MS (10) HC (10) | BRAZIL | Expression of HERV-Wenv MS 90% HC 50% | ***do Olival 2013^16^*** |
| HERV-Wenv DNA/RNA/protein syncytin-1 DNA/RNA MSRVenv DNA/RNA | PLASMA  CULTURE MEDIUM PBMC | RT-qPCR WB | MS (8) Blood donors (8) | SARDINIA (ITALY) | Increased expression of MSRVenv  in MS (p=0.02) No difference in expression of HERV-Wenv and sincytin-1 | ***Mameli 2009^10^*** |
| HERV-W/MSRVenv RNA | PLASMA | RT-qPCR | MS (149) OND (77) HC (98) | EUROPE (SWEDEN, SPAIN, ITALY, SARDINIA) | Increased expression of MSRVenv in plasma and CSF in MS compared with HC (p<0.001) and OND (p<0.05) | ***Arru 2007^17^*** |
|  | CSF |  | MS (98) OND (81) |  |  |  |
| HERV-W/MSRVpol RNA | PLASMA | nested PCR | MS (149) OND (77) HC (98) |  | Expression of MSRVpol in PLASMA MS 71.4%  OND 40.3% HC 17.3% |  |
|  | CSF |  | MS (98) OND (81) |  | Expression of MSRVpol in CSF MS 80.6%  OND 40% |  |
| HERV-W/MSRVenv RNA | PBMC | RT-qPCR | MS (35) Controls (14) | SARDINIA (ITALY) | Expression of MSRVenv PBMC MS 100%  HC 28.6%  Increased expression of HERV-W/MSRV env copy number in brain and PBMC of MS compared with OND (p=0.014) and HC (p=0.006) | ***Mameli 2007^18^*** |
|  | BRAIN | RT-qPCR | PrMS (3) OND (8) HC (4) |  |  |  |
| HERV-W/MSRV pol RNA |  | RT-PCR |  |  | Increased expression of HERV-W/MSRV pol in brain of MS compared with HC (p=0.017) |  |
| HERV-W/MSRV pol RNA | PLASMA | RT-PCR | MS (39) OND (35) HC (39) | SARDINIA (ITALY) | Expression of MSRVpol in plasma MS 100%  OND 42.9% HC 12.8% | ***Dolei 2002^20^*** |
|  | CSF |  | MS (31) OND (10) |  | Expression of MSRVpol in CSF MS 80.6%  OND 40% |  |
| MSRVpol RNA | PLASMA | RT-PCR | MS (25) HC (25) | SARDINIA (ITALY) | Expression of MSRVpol in plasma MS 100% HC 12% | ***Serra 2001^21^*** |
| HERV-W ENV protein | BRAIN | IHC | MS brain (20)  Controls (6) | NETHERLANDS | HERV-W ENV detected in lesions of all MS brains. HERV-W expression not observed in control brains. | ***Van Horssen 2016^32^*** |
| MSRVenv RNA | PBMC | RT-qPCR | MS (58) HC (26) | EUROPE | Expression of MSRVenv RNA MS 40%  HC 4% | ***Perron 2012^19^*** |
| MSRVenv DNA copy number |  |  | MS (62) HC (26) |  | MSRVenv DNA copy number MS 40% HC 4% |  |
| HERV-W ENV protein | SERUM | ELISA | MS (74) CIS (14) HC (26) |  | Expression of MSRV ENV PROTEIN MS 80%  CIS 64%  HC 0% |  |
| HERV-W ENV protein | BRAIN | IHC | MS chronic active lesions (5) MS early active regions (3) NAWM and non-neuro (3) |  | 100% MS brain lesions positive  NAWM and non-MS brains negative |  |
| MSRV/HERV-Wgag  MSRV/HERV-Wenv | BRAIN | IHC | MS brain (24) controls (18) | EUROPEAN (FRANCE, AUSTRIA, GERMANY) | “MS-specific” GAG and ENV patterns in MS lesions at the level of  endothelial and microglial cells | ***Perron 2005^31^*** |
| MSRVpol RNA | SUPERNATANT MMCS | RT-PCR ELOSA | MS (4) HC (1) | FRANCE | Expression of MSRVpol MS 50% HC 0% | ***Menard 1997^22^*** |
| HERV-W/MSRV pol RNA | SERUM | retroPCR | MS (17) HC (44) MS treated (6) MS untreated (15) | FRANCE | Expression of MSRVpol MS 53% HC 7% MS untreated 100% MS treated 27% | ***Garson 1998^8^*** |
| HERV-W/MSRV pol RNA | CSF PLASMA | retroPCR  ELOSA | MS (10) OND (10) | FRANCE | Expression of MSRVpol 50% 0% | ***Perron 1997^5^*** |
| HERV-W ENV TM ans SU protein | PBMC | FC | active MS (23) nonactive MS (23) HC (22) epilepsy (11) | DENMARK | Increased expression of HERV-W ENV in CD19^+^cells (p<0.001) and CD14^+^ cells (p<0.05) in active MS patients compared to stable MS and controls. No detection in CD4^+^ and CD8^+^ T cells in any group | ***Brudek 2009^26^*** |
| syncytin-1 DNA RNA MSRVenv DNA RNA | BRAIN | RT-qPCR | MS (20) OND (19) | CANADA | Increased syncytin-1 DNA (P<0.05) and RNA (P<0.01) in MS compared with OND.  No differences in MSRVenv expression | ***Antony 2007^7^*** |
| syncytin-1DNA MSRVenv DNA | PBMC |  | MS (33) UNAFFECTED FAMILY MEMBERS (33) |  | No difference in the detection of either syncytin-1 or MSRVenv DNA in PBMC |  |
| syncytin-1RNA MSRVenv RNA | CSF PLASMA |  | MS (40) OND(38) |  | No difference in the expression of either syncytin-1 or MSRVenv RNA in CSF and plasma |  |
| syncytin-1 RNA and DNA copy number | BRAIN PBMC CSF PLASMA | RT-qPCR | MS (20) OND (19) | CANADA | Increased RNA expression (p<0.01), RNA (p<0.05) and DNA (p<0.001) copy number of syncytin-1 in MS brain compared to controls, but not in B cells, T cells, monocytes, plasma and CSF | ***Antony 2006^28^*** |
| HERV-Wenv syncytin-1 RNA | BRAIN | RT-qPCR | MS (14) OND (11) | CANADA | Increased expression of HERV-Wenv in MS compared to OND (p<0.01) by RT-PCR | ***Antony 2004^33^*** |
| SYNCYTIN-1 PROTEIN |  | WB IHC | MS (16) OND (18) |  | Increased expression of SYNCYTIN-1 in MS compared to OND (p<0.05) by WB |  |
| HERV-Wpol RNA | BRAIN | RT-PCR Southern Blot | PrMS (6) Alzheimer`s disease (6) HIV (6) | CANADA | HERV-W RNA level increased in MS (p < 0.001) and HIV-infected (p < 0.005) patients compared with controls | ***Johnston 2001^34^*** |
| HERV-W/MSRV pol RNA | SERUM | RT-PCR | MS(49) Healthy close relatives (20) Controls (39) | SOUTH AFRICA (EUROPEAN DESCENT) | Expression of MSRVpol  MS 69% Healthy close relatives 70% HC 0% | ***de Villiers 2006^23^*** |
| HER-W/MSRVpol  DNA copy number | PBMC | FISH | MS (16) Controls (10) | POLAND | MS average value ranged 6.18-24.02 HC average value ranged 3.22-6.48 | ***Zawada 2003^24^*** |
| HERV-W/MSRV pol RNA | SERUM | PCR FISH | MS (32) Myasthenia gravis (17) Parkinson`s disease (16) Migraine (21) HC (27) | POLAND | Expression of MSRVpol RNA MS 96.9%  MG 52.9%  PD 43.8%  MI 47.6%  HC 48.1% | ***Nowak 2003^25^*** |
| HERV-W/MSRV pol RNA | PBMC |  |  |  | Expression of MSRVpol RNA MS 100%  MG 70.6%  PD 56.3%  MI 66.7%  HC 66.7% |  |
| HERV-W/MSRV pol DNA |  |  |  |  | Detection of MSRVpol DNA MS 100%  MG 100%  PD 100%  MI 100%  HC 100% |  |
| HERV-W/MSRV pol RNA | CSF |  | MS (3) |  | Expression of MSRVpol RNA MS 100% |  |

**Gray shading indicates studies that did not find an association between HERV-W and MS.*

*Pol,* Polymerase; *env*, Envelope; *gag,* Group specific antigen; *PBMC,* Peripheral Blood Mononuclear Cells; *CSF, C*erebrospinal Fluid; *M/M*, Monocyte/Macrophages; *RT-PCR*, Reverse Transcription Polymerase Chain Reaction; *NGS, N*ext Generation Sequencing; *FC,* Flow Cytometry; *WB,* Western Blot; *ELISA,* Enzyme-Linked Immunosorbent Assay; *IHC*, Immunohistochemistry; *ELOSA,* Enzyme-Linked Oligosorbent Assay; *FISH,* Fluorescence In Situ Hybridization; *MS,* Multiple Sclerosis; *HC*, Healthy Control; *OND,* Other Neurological Disease; *RRMS,* Relapsing-Remitting MS; *PrMS,* Progressive MS; *NAWM*, Normal Appearing White Matter.
