# Supplementary material for: The association between human endogenous retroviruses and multiple sclerosis: A systematic review and meta-analysis
Source: PLoS One. 2017 Feb 16;12(2):e0172415. doi: 10.1371/journal.pone.0172415 (PMC5313176; doi:10.1371/journal.pone.0172415)
Supplement: S3 Table — (DOCX) [file pone.0172415.s003.docx]

S3 Table

| **HERV** | **SAMPLE** | **TECHNIQUE** | **POPULATION IN THE STUDY** | **COUNTRY** | **RESULT** | **STUDY** |
| --- | --- | --- | --- | --- | --- | --- |
| HERV-Fc1 DNA (SNP rs391745 ) | PBMC | PCR mass spectrometry | MS (350)  HC (40) | DENMARK | Interaction of SNPs near HERV-Fc1 rs391745, HERV-K13 rs2435031, and HLA  rs2135388 associated with MS | ***Nexo 2016^39^*** |
| HERV-Fc1 DNA copy number | PBMC | nested PCR Southern blot | MS (74) HC (7) | DENMARK | No additional germ line copies  of HERV-Fc1 identified | ***Nissen 2012^42^*** |
| HERV-Fc1 gag RNA | PLASMA | RT-qPCR | active MS (22) non-active MS (19) HC (30) | DENMARK | Increased expression of HERV-Fc1 RNA in active MS patients (p<0.01) compared with non-active MS and HC | ***Laska 2012^41^*** |
| HERV-Fc1gag DNA copy number | PBMC | RT-qPCR |  |  | No difference in DNA HERF-Fc1gag copy number |  |
| HERV-H/F GAG protein | PBMC | FC |  |  | HERV-Fc1 GAG protein increased in active MS patients in CD4^+^ (p=0.01), CD8^+^ (p=0.01) cells and in active (p=0.036) and non-active (p=0.035) MS patients in monocytes compared with HC |  |
| HERV-Fc1 DNA (SNP rs391745 ) | TOTAL BLOOD | PCR mass spectrometry | BOMS (941) PPMS (140) HC (1838) | DENMARK NORWAY | Detection of HERV-Fc1 BOMS 18% PPMS 14% HC 14 % | ***Hansen 2011^40^*** |
| HERV-Fc1 DNA (SNP rs391745 ) | TOTAL BLOOD | PCR mass spectrometry | MS (1062) HC (2082) | DENMARK | Detection of HERV-Fc1 MS 20% HC 14 % | ***Nexo 2011^38^*** |
| HERV-H ENV TM and SU protein | PBMC | FC | active MS (23) non-active MS (23) HC (22) epilepsy (11) | DENMARK | Increased expression of HERV-H ENV in CD19^+^ cells (p<0.001) and CD14^+^ cells (p<0.05) in active MS patients compared with stable MS and controls. No detection in CD4^+^ and CD8^+^ T cells in any group | ***Brudek 2009^26^*** |
| HERV-H RGH-2 env/pro RNA | PBMC | RT-PCR | MS (37) Alzheimer’s disease (15) HC (16) | DENMARK | Expression of HERV-H MS 38% AD 7% HC 13% | ***Christensen 2003^37^*** |
| HERV-Hgag RGH RNA | PLASMA | RT-PCR | MS (33) Alzheimer’s disease (29) HC (20) | DENMARK | Expression of HERV-H MS 73% AD 0% HC 0% | ***Christensen 2000^36^*** |
| HERV-Fc1 DNA  (SNP rs391745 ) | PBMC | RT-qPCR | MS (2473) HC (3031) | SPAIN | Detection of HERV-Fc1 MS 17.9% HC 17.7% | ***de la Hera 2014^43^*** |
| HERV-H DNA and RNA | CSF | RT-qPCR | MS (48) inflammatory OND (23) no inflammatory OND (21) | SPAIN | Expression of HERV-H RNA and DNA detection MS 0% inflammatory OND 0% no inflammatory OND 0% | ***Alvarez-Lafuente 2008^30^*** |
| HERV-Henv RNA | BRAIN PBMC | RT-qPCR | RR-MS (9) CONTROLS (9) | CANADA | No difference in the expression of HERV-H | ***Antony 2006^28^*** |
| HERV-Henv RNA | BRAIN | RT-qPCR | MS (14) OND (11) | CANADA | No difference in the expression of HERV-H | ***Antony 2004^33^*** |
| HERV-Hgag RNA | BRAIN | RT-PCR Southern Blot | PrMS (6) Alzheimer’s disease (6) HIV (6) | CANADA | No difference in the expression of HERV-H | ***Johnston 2001^34^*** |

**Gray shading indicates studies that did not find an association between HERV-H and MS.*

*SNP*, single nucleotide polymorphism; *env*, Envelope; *gag,* Group specific antigen; *PBMC,* Peripheral Blood Mononuclear Cells; *CSF, C*erebrospinal Fluid; *RT-PCR*, Reverse Transcription Polymerase Chain Reaction; *FC,* Flow Cytometry; *MS,* Multiple Sclerosis; *HC*, Healthy Control; *OND,* Other Neurological Disease; *RRMS,* Relapsing-Remitting MS; *BOMS,* bout onset MS; *PPMS,* Primary Progressive MS;
