# Supplementary material for: The association between human endogenous retroviruses and multiple sclerosis: A systematic review and meta-analysis
Source: PLoS One. 2017 Feb 16;12(2):e0172415. doi: 10.1371/journal.pone.0172415 (PMC5313176; doi:10.1371/journal.pone.0172415)
Supplement: S4 Table — (DOCX) [file pone.0172415.s004.docx]

S4 Table

| **HERV** | **SAMPLE** | **TECHNIQUE** | **POPULATION IN THE STUDY** | **COUNTRY** | **RESULT** | **STUDY** |
| --- | --- | --- | --- | --- | --- | --- |
| HERV-K133 (SNP rs2435031 ) | PBMC | PCR mass spectrometry | MS (350)  HC (40) | DENMARK | Interaction of SNPs near HERV-Fc1 rs391745, HERV-K13 rs2435031, and HLA  rs2135388 associated with MS | ***Nexo 2016^39^*** |
| HERV-K18env DNA | BLOOD | RT-qPCR | MS (942) Rheumatoid arthritis (462) HC (601) | SPAIN | Increased detection of HERV-K18.3 in MS (p=0.02) | ***De la Hera 2013^48^*** |
| HERV-K18env DNA | BLOOD | RT-qPCR | MS (207 replicated 909) HC (403 replicated 339) | USA | Increased detection of HERV-K18.3 in MS (p=0.03) | ***Tai 2008^49^*** |
| HERV-K113 DNA | BLOOD | PCR | MS (951) UNAFFECTED PARENTS (1902) | UK | Detection of HERV-K113  MS 7.36% UP 6.52% | ***Moyes 2008^47^*** |
| HERV-K113 DNA HERV-K115 DNA | BLOOD | PCR | MS (109) Rheumatoid arthritis (96) Sjögren’s syndrome (96) HC (96) | UK | Detection of HERV-K113 HERV-K115 MS 11.9% MS4.4  RA 5.7% RA 5.2% SS 15.6% SS 0%  HC 4.2 % HC 1% | ***Moyes 2005^46^*** |
| HERV-Kenv RNA | BRAIN PBMC | RT-qPCR | RR-MS (9) CONTROLS (9) | CANADA | No difference in the expression of  HERV-Kenv RNA | ***Antony 2006^28^*** |
| HERV-Kenv RNA | BRAIN | RT-qPCR | MS (14) OND (11) | CANADA | No difference in the expression of  HERV-Kenv RNA | ***Antony 2004^33^*** |
| HERV-Kpol RNA | BRAIN | RT-PCR Southern Blot | PrMS (6) Alzheimer’s disease (6) HIV (6) | CANADA | HERV-K RNA level increased in MS (p < 0.01) and HIV-infected (p < 0.05) patients compared with controls | ***Johnston 2001^34^*** |
| HERV-K10env RNA | PBMC BRAIN | PCR | MS (22) HC (22) OND (5) | FRANCE | Expression of HERV-K10env RNA  MS 100% HC 100% OND 100% | ***Rasmussen 1997^45^*** |

**Gray shading indicates studies that did not find an association between HERV-K and MS.*

*SNP*, single nucleotide polymorphism; *env*, Envelope; *pol,* Polymerase; *PBMC,* Peripheral Blood Mononuclear Cells; *RT-PCR*, Reverse Transcription Polymerase Chain Reaction; *MS,* Multiple Sclerosis; *HC*, Healthy Control; *OND,* Other Neurological Disease; *RRMS,* Relapsing-Remitting MS; *PrMS,* Progressive MS; *RA,* Rheumatoid arthritic; *SS*, Sjögren’s syndrome
