# Supplementary material for: The association between human endogenous retroviruses and multiple sclerosis: A systematic review and meta-analysis
Source: PLoS One. 2017 Feb 16;12(2):e0172415. doi: 10.1371/journal.pone.0172415 (PMC5313176; doi:10.1371/journal.pone.0172415)
Supplement: S5 Table — 1 article studying the association between HERV-15 and MS by 1 research group. (DOCX) [file pone.0172415.s005.docx]

S5 Table

| **HERV** | **SAMPLE** | **TECHNIQUE** | **POPULATION IN THE STUDY** | **COUNTRY** | **RESULT** | **STUDY** |
| --- | --- | --- | --- | --- | --- | --- |
| HRES-1 DNA polymorphism | BLOOD | Genotyping by enzymatic reaction | MS (110) HC (100) | UK | Association between HRES-1 haplotype 1 and MS (p < 0.01) | ***Rasmussen 2000^50^*** |
| HRES-1 DNA polymorphism | BLOOD | SSCP SEQUENCING | MS (78) HC (122) | DENMARK | Association between HRES-1 haplotype 2 and 3 and MS (p=0.03)  Inverse correlation between HERES-1 haplotype 1 and 4 and MS | ***Rasmussen 1999^51^*** |
| HRES-1 DNA polymorphism | BLOOD | Genotyping by enzymatic reaction | MS (42) HC (88) | CHINA | No difference in the haplotypes between MS and HC | ***Rasmussen 1998^53^*** |
| HRES-1 RNA | PBMC BRAIN | PCR | MS (22) HC (22) OND (5) | FRANCE DENMARK | Expression of HRES-1 RNA  MS 68% HC 73% OND 80% | ***Rasmussen 1997^45^*** |
| HRES-1 DNA polymorphism | BLOOD | PCR SSCP | MS (87) HC (158) | DENMARK | Association between HRES-1 haplotype 2 and 3 and MS (p=0.014). Inverse correlation between HERES-1 haplotype 1 and MS | ***Rasmussen 1996^52^*** |
| **HERV** | **SAMPLE** | **TECNIQUE** | **POPULATION IN THE STUDY** | **COUNTRY** | **RESULT** | **STUDY** |
| HERV-15 | TOTAL BLOOD | GWAS | PrMS (197) HC (234) | ITALY AUSTRALIA NORTHERN EUROPE | Association with a locus on chromosome 7q35 [rs996343(G)], which maps within a human endogenous retroviral (HERV) element | ***Martinelli-Boneschi 2012^54^*** |

**Gray shading indicates studies that did not find an association between HRES-1 and MS.*

*PBMC,* Peripheral Blood Mononuclear Cells; *SSCP*, Single-strand Conformation Polymorphism; *PCR*, Polymerase Chain Reaction; *GWAS*, Genome-Wide Association Study; *MS,* Multiple Sclerosis; *HC,* Healthy Control; *OND,* Other Neurological Disease; *PrMS,* Progressive MS.
